# Food Insecurity, Physical Activity, and Psychological Factors as Predictors of Life Satisfaction in Older Adults

**DOI:** 10.3390/geriatrics11050131

**Published:** 2026-09-14

**Authors:** Mónica Lorena Hernández-Trujillo, Wilter C. Morales-García, Mardel Morales-García

**Affiliations:** 1 Public Health Graduate Unit, Graduate School, Universidad Peruana Unión, Lima 15464, Peru; monica.hernandez@upeu.edu.pe; 2 Faculty of Theology, Universidad Peruana Unión, Lima 15464, Peru; wiltermorales@upeu.edu.pe; 3 Research Sub-Directorate, Graduate School, Universidad Peruana Unión, Lima 15464, Peru

**Keywords:** life satisfaction, older adults, perceived stress, physical activity, food insecurity, subjective well-being

## Abstract

**Background:** Life satisfaction is a key component of subjective well-being in later life and may be associated with psychological, economic, family, functional, and food-related factors. **Objective:** This study aimed to identify factors associated with life satisfaction among older adults, considering sociodemographic characteristics, food insecurity, physical activity, and psychological factors. **Methods:** A quantitative, observational, analytical, cross-sectional study was conducted with 200 noninstitutionalized older adults from Sogamoso, Colombia. Self-report instruments assessed life satisfaction, food insecurity, physical activity, depression, anxiety, and perceived stress. Data were analyzed using descriptive statistics, group comparison tests, Pearson correlations, and multiple linear regression. **Results:** Higher perceived stress (*B* = −0.762, *p* < 0.001), perceived insufficient income (*B* = −3.380, *p* < 0.001), self-employment (*B* = −2.153, *p* = 0.009), other sources of income (*B* = −2.569, *p* = 0.017), and technical education (*B* = −4.744, *p* = 0.003) were associated with lower life satisfaction. Conversely, combined engagement in strength and flexibility exercises (*B* = 3.203, *p* < 0.001), having two children (*B* = 3.498, *p* = 0.002), and having three or more children (*B* = 2.618, *p* = 0.008) were associated with higher life satisfaction. Primary education was not significantly associated with life satisfaction after adjustment (*p* = 0.093). Food insecurity was associated with life satisfaction in the bivariate analysis but did not remain statistically significant in the adjusted model. The regression model explained 48.7% of the variance in life satisfaction (*R*^2^ = 0.487; adjusted *R*^2^ = 0.457). **Conclusions:** Life satisfaction among older adults was associated with psychological, economic, family, and functional factors. The findings highlight the relevance of perceived stress, economic security, functional physical activity, and family resources when addressing subjective well-being in later life.

## 1. Introduction

Life satisfaction is a central dimension of subjective well-being, understood as the overall cognitive evaluation a person makes of the quality of their life according to their own criteria [1,2]. Unlike transient emotional states, life satisfaction reflects a relatively stable assessment of personal well-being and is related to the perception of achievement, meaning in life, health, available resources, and the quality of social relationships [3,4]. In old age, this evaluation becomes especially relevant because it integrates functional, economic, family, psychological, and contextual conditions that may either promote or undermine the experience of well-being.

The study of life satisfaction in older adults is situated within the contemporary framework of healthy aging, which is not limited to the absence of disease but emphasizes functional capacity, autonomy, social participation, and the possibility of living in accordance with what the person values [5]. This perspective is particularly relevant in Latin American contexts, where many older adults face economic inequalities, food insecurity, labor informality, limited access to services, family dependence, and heterogeneous support networks. Therefore, understanding the factors associated with life satisfaction at this stage of life requires a multidimensional approach that integrates both individual resources and social conditions.

From a theoretical perspective, the present study is grounded primarily in the conservation of resources theory, which proposes that individuals seek to acquire, maintain, and protect valuable resources, and that stress emerges when those resources are lost, threatened, or not recovered after a significant investment [6]. From this perspective, variables such as income, housing, food security, physical activity, mental health, and family support can be understood as resources or threats that influence the overall evaluation of life. In older adults, the loss or fragility of these resources may have a cumulative effect on well-being, especially when unfavorable economic conditions are combined with psychological distress.

Sociodemographic variables constitute a first explanatory level of life satisfaction in old age. Sex and age have shown variable associations with subjective well-being due to differences in health trajectories, social roles, access to resources, and life expectations [7,8]. Marital status may also influence life satisfaction, since living with a partner or experiencing widowhood, singlehood, or divorce can modify sources of emotional and practical support [9]. Likewise, the number of children may represent a potential source of companionship, family support, and sense of purpose, although its effect depends on the quality of relationships and the social context [10,11,12].

Educational level, area of residence, type of housing, source of income, and perceived economic sufficiency are also relevant for understanding life satisfaction. Higher education is often associated with better employment opportunities, greater access to information, better health conditions, and broader social networks [13,14]. Similarly, economic and housing conditions may affect the perception of security, autonomy, and control over daily life. Evidence indicates that subjective financial satisfaction may be even more relevant to well-being than objective income, especially when individuals evaluate their ability to meet basic needs [14,15]. In older adults, this perception becomes increasingly important due to reliance on pensions, family income, self-employment, or informal support.

Food insecurity is another relevant social determinant of well-being in old age, as it compromises not only nutrition, but also security, autonomy, and emotional stability. Recent evidence shows that food insecurity is associated with greater deterioration in mental health among older adults, particularly in low- and middle-income countries [16]. This relationship may be explained by increased depressive, anxious, and stress-related symptoms, as well as by feelings of uncertainty, dependence, or shame linked to difficulties in securing daily food [17,18]. For this reason, food insecurity should be understood not only as a nutritional indicator, but also as a psychosocial condition that may affect the overall evaluation of life.

Physical activity represents a protective resource for well-being in older adults. Various studies have shown that regular physical activity is associated with better mental health, greater functionality, reduced depressive and anxiety symptoms, and a better perceived quality of life [19,20,21]. In particular, activities aimed at strength, balance, and flexibility may be especially important in old age because of their relationship with mobility, functional independence, and the prevention of physical limitations. It has also been reported that functional fitness may mediate the relationship between physical activity and life satisfaction in community-dwelling older adults [22]. From the perspective of the conservation of resources theory, physical activity can be interpreted as a resource that strengthens autonomy, self-efficacy, and perceived control.

Psychological factors, such as stress, anxiety, and depression, play a fundamental role in life satisfaction. The presence of psychological distress has been consistently associated with lower levels of subjective well-being, whereas low life satisfaction may increase vulnerability to emotional symptoms, forming a potentially bidirectional relationship [23]. In older adults, this association is especially important because emotional symptoms may be underdetected or mistakenly attributed to normal aging. In addition, stress may operate as a central mechanism through which economic, food-related, functional, and social difficulties undermine the overall evaluation of life.

Despite the existing evidence, further research is still needed to simultaneously integrate sociodemographic factors, food insecurity, physical activity, and psychological distress as predictors of life satisfaction in Latin American older adults. Many studies have examined these factors in isolation, but analyses that incorporate them into a single explanatory model are less common, especially in noninstitutionalized older populations. Therefore, the objective of the present study was to identify significant predictors of life satisfaction in older adults, considering sociodemographic variables, food insecurity, physical activity, and psychological factors such as stress, anxiety, and depression.

## 2. Materials and Methods

### 2.1. Study Design

A quantitative, observational, analytical, cross-sectional study was conducted to identify predictors of life satisfaction in noninstitutionalized older adults. The reporting of the study followed the recommendations of the STROBE guidelines for observational studies [24].

### 2.2. Participants

The population consisted of older adults residing in the municipality of Sogamoso, Colombia. The final sample included 200 participants aged 60 years or older, selected through nonprobability convenience sampling. Non-institutionalized older adults were included if they voluntarily agreed to participate in the study and had sufficient physical, auditory, visual, and cognitive conditions to complete the questionnaire. Individuals were excluded if they stated that they did not wish to participate, provided incomplete responses, or reported neurological conditions that could compromise their understanding of the instrument, such as Parkinson’s disease, Huntington’s disease, brain tumors, hydrocephalus, epilepsy, subdural hematoma, multiple sclerosis, or a history of stroke.

The minimum sample size was estimated using G*Power 3.1.9.7 [25]. For a multiple linear regression with 14 predictors, a medium effect size (f^2^ = 0.15), statistical power of 0.80, and a significance level of 0.05 were assumed. Under these parameters, the minimum required sample size was 135 participants. Considering potential losses or incomplete questionnaires, 202 responses were collected; of these, 2 were excluded due to incomplete data, resulting in a final analysis sample of 200 participants.

### 2.3. Procedure

To access the study population, community leaders and representatives of primary care centers in the municipality of Sogamoso, Colombia, were contacted. They were informed about the purpose of the research and asked to support dissemination of the questionnaire. The instrument was administered through an online form created using Google Forms (Google LLC, Mountain View, CA, USA; https://docs.google.com/forms, accessed on 18 July 2025) and shared primarily through social media and instant messaging platforms, including WhatsApp (WhatsApp LLC, Menlo Park, CA, USA; https://www.whatsapp.com, accessed on 18 July 2025). Because WhatsApp was accessed through different devices and automatically updated versions, no single controlled software version was applicable. This data collection modality was used because of its operational feasibility and community reach, following ethical recommendations for Internet-based research [26].

Before completing the questionnaire, participants received information about the objective of the study, the voluntary nature of their participation, the confidentiality and anonymity of their responses, and their right to withdraw at any time without any consequences. Only those who provided informed consent proceeded to the questionnaire. No personal data that could directly identify participants were collected.

### 2.4. Instruments

Sociodemographic data. A self-report form was used to collect information on sex, age, marital status, number of children, educational level, area of residence, current source of income, and perceived income sufficiency to meet basic needs. These variables were included because of their relevance as indicators of social, economic, and family resources associated with well-being in old age.

*Food insecurity*. The Food Insecurity Experience Scale (FIES), developed by the Food and Agriculture Organization of the United Nations (FAO), was used. The FIES is an experience-based measure that assesses limited access to adequate food in terms of quantity and quality through eight items reflecting different levels of food insecurity severity [27,28]. In this study, participants were classified as having mild, moderate, or severe food insecurity according to their score. In the present sample, the FIES showed good internal consistency (KR-20 = 0.864).

*Physical activity*. Physical activity was assessed using the Rapid Assessment of Physical Activity (RAPA), a brief instrument designed to rapidly evaluate physical activity levels in older adults [29]. The RAPA identifies levels of aerobic activity, and additionally, the performance of strength and flexibility exercises—dimensions that are especially relevant in older populations because of their relationship with functionality, mobility, and independence. The Spanish version has shown adequate reliability and usefulness for the rapid assessment of physical activity in older adults [30]. An internal consistency coefficient was not estimated in the present study because the RAPA assesses distinct physical activity domains and is not intended to function as a unidimensional reflective scale.

*Depression*. Depressive symptomatology was assessed using the Patient Health Questionnaire-2 (PHQ-2), an ultra-brief instrument composed of two items that explore the presence of depressed mood and anhedonia during the previous two weeks. The PHQ-2 was developed as an initial screening tool for depression and has shown adequate evidence of validity in clinical and community settings [31]. In the Colombian population, acceptable psychometric properties have also been reported for its use as a brief measure of depressive symptoms [32]. In the present sample, the PHQ-2 showed a Cronbach’s alpha of 0.656.

*Anxiety*. Anxiety was measured using the Generalized Anxiety Disorder-2 (GAD-2), a brief two-item scale derived from the GAD-7 and designed to screen for core symptoms of generalized anxiety. The GAD-2 has demonstrated usefulness for the rapid detection of anxiety symptoms in primary care and population-based studies [33]. The Spanish version has reported adequate psychometric indicators, including high internal consistency [34]. In the present sample, the GAD-2 showed good internal consistency (Cronbach’s α = 0.816).

*Perceived stress*. Stress was assessed using the four-item Perceived Stress Scale (PSS-4), a brief version of the scale developed to measure the subjective perception of stress during the past month [35]. The PSS-4 assesses the extent to which individuals perceive life situations as unpredictable, uncontrollable, or overwhelming. Previous studies have reported adequate psychometric properties for this brief version in Latin American populations, with alpha and omega coefficients [36]. In the present sample, the PSS-4 showed a Cronbach’s alpha of 0.601.

*Life satisfaction*. The dependent variable was assessed using the Satisfaction With Life Scale (SWLS), developed by Diener et al. [37]. This scale consists of five items that assess the global cognitive judgment a person makes about their own life. Responses are recorded on a seven-point Likert-type scale, with higher scores indicating greater life satisfaction. The SWLS has shown adequate indicators of internal consistency, temporal stability, and convergent validity across different populations [38]. In the present sample, the SWLS showed good internal consistency (Cronbach’s α = 0.826).

### 2.5. Statistical Analysis

Data were analyzed using R software [39]. Statistical analyses were conducted using the R programming environment (version 4.6.1; 2026) within the RStudio integrated development environment (version 2026.08.1; 2026). First, descriptive analyses were performed for sociodemographic characteristics, physical activity, food insecurity, psychological symptoms, and life satisfaction. Absolute frequencies and percentages were calculated for categorical variables, whereas means and standard deviations were calculated for continuous variables.

Internal consistency was assessed for the instruments used in the study. Cronbach’s alpha was calculated for the PHQ-2, GAD-2, PSS-4, and SWLS, whereas the Kuder–Richardson coefficient (KR-20) was used for the dichotomous items of the FIES.

Bivariate analyses were subsequently conducted to compare life satisfaction scores according to the participants’ characteristics. Welch’s independent-samples *t*-test was used for categorical variables with two groups. For variables with three or more categories, one-way analysis of variance (ANOVA) was performed. When statistically significant differences were identified, Tukey’s honestly significant difference (HSD) test was used for post hoc comparisons. Pearson’s correlation coefficient was used to examine the associations between depression, anxiety, perceived stress, and life satisfaction.

Finally, multiple linear regression analysis was performed to identify factors independently associated with life satisfaction. The total SWLS score was entered as the dependent variable. The predictors included perceived stress and selected sociodemographic, economic, food insecurity, and physical activity variables. Categorical predictors were represented by dummy variables corresponding to the categories included in the regression model, with the omitted categories constituting the respective reference groups.

The assumptions of multiple linear regression were examined, including linearity, independence of errors, normality and homoscedasticity of residuals, absence of problematic multicollinearity, and influential observations. Multicollinearity was assessed using the variance inflation factor (VIF), with values below 5 considered acceptable [40]. Statistical significance was established at *p* < 0.05.

### 2.6. Ethical Considerations

The study was approved by the Ethics Committee of Universidad Peruana Unión (approval code 2025-CE-EPG-00107; 9 June 2025). The research was conducted in accordance with the ethical principles of the Declaration of Helsinki for research involving human participants [41]. Participation was voluntary, anonymous, and confidential. Before completing the questionnaire, all participants received information about the purpose of the study, the voluntary nature of participation, confidentiality of their responses, and their right to withdraw at any time without consequences. Electronic informed consent was obtained from all participants before access to the questionnaire was granted.

## 3. Results

### 3.1. Sociodemographic Characteristics, Physical Activity, and Food Insecurity According to Life Satisfaction

Table 1 presents the life satisfaction scores according to sociodemographic characteristics, physical activity, and food insecurity. Statistically significant differences were identified according to perceived income sufficiency, educational level, marital status, type of income, engagement in strength and flexibility exercises, and food insecurity level. Older adults who reported having sufficient income had higher life satisfaction scores (M = 26.89, SD = 5.46) than those who perceived their income as insufficient (M = 21.34, SD = 5.96), with a statistically significant difference, *t*(194.83) = −6.87, *p* < 0.001. Significant differences were also observed according to educational level, *F*(4, 195) = 5.91, *p* < 0.001; marital status, *F*(4, 195) = 5.24, *p* < 0.001; type of income, *F*(3, 196) = 9.54, *p* < 0.001; strength and flexibility practice, *F*(3, 196) = 8.00, *p* < 0.001; and food insecurity, *F*(2, 197) = 11.48, *p* < 0.001.

Tukey post hoc comparisons showed that participants with graduate studies reported higher life satisfaction than those with a high school education (*p* < 0.001) and those with primary education (*p* < 0.001). Importantly, no significant difference was observed between participants with primary education and those with a high school education (*p* = 0.9998). Married participants reported higher life satisfaction than divorced (*p* = 0.010) and single participants (*p* = 0.002). Pensioners reported higher life satisfaction than employees (*p* = 0.010), self-employed participants (*p* = 0.008), and those with other sources of income (*p* < 0.001). Participants who practiced both strength and flexibility exercises reported higher life satisfaction than those who performed neither type of exercise (*p* < 0.001) and those who performed strength exercises only (*p* = 0.005). Finally, participants with mild food insecurity showed higher life satisfaction than those with moderate (*p* < 0.001) or severe food insecurity (*p* = 0.007), whereas the moderate and severe groups did not differ significantly (*p* = 0.953). In contrast, no statistically significant differences in life satisfaction were found according to sex, *t*(160.34) = −1.26, *p* = 0.209; area of residence, *t*(45.86) = 1.38, *p* = 0.175; age, *F*(2, 197) = 0.28, *p* = 0.753; number of children, *F*(3, 196) = 2.13, *p* = 0.098; or aerobic activity level, *F*(4, 195) = 2.37, *p* = 0.054.

### 3.2. Correlations Between Depression, Anxiety, Stress, and Life Satisfaction

Table 2 shows the means, standard deviations, and correlations between psychological variables and life satisfaction. Life satisfaction showed negative and statistically significant correlations with depression (r = −0.31, *p* < 0.01), anxiety (r = −0.41, *p* < 0.01), and perceived stress (r = −0.52, *p* < 0.01). These results indicate that higher levels of psychological distress were associated with lower levels of life satisfaction.

Significant positive correlations were also observed among the psychological variables. Depression was positively related to anxiety (r = 0.57, *p* < 0.01) and perceived stress (r = 0.52, *p* < 0.01). Similarly, anxiety showed a strong positive correlation with perceived stress (r = 0.71, *p* < 0.01).

### 3.3. Predictors of Life Satisfaction

A multiple linear regression model was conducted to identify predictors of life satisfaction in older adults. The model included perceived stress and the dummy-coded sociodemographic, economic, food insecurity, and physical activity variables shown in Table 3. The overall model was statistically significant, *F*(11, 188) = 16.23, *p* < 0.001, explaining 48.7% of the variance in life satisfaction (*R*^2^ = 0.487; adjusted *R*^2^ = 0.457). No evidence of problematic multicollinearity was observed, with all variance inflation factor values below 2.1. Perceived stress was negatively associated with life satisfaction (*B* = −0.762, SE = 0.112, *t* = −6.805, *p* < 0.001), indicating that higher levels of perceived stress were associated with lower life satisfaction. Perceived insufficient income was also negatively associated with life satisfaction (*B* = −3.380, SE = 0.774, *t* = −4.366, *p* < 0.001). Regarding source of income, self-employment (*B* = −2.153, SE = 0.814, *t* = −2.646, *p* = 0.009) and other sources of income (*B* = −2.569, SE = 1.063, *t* = −2.417, *p* = 0.017) were negatively associated with life satisfaction compared with pension income, whereas employment income was not statistically significant (*B* = −2.054, *p* = 0.121).

In contrast, combined engagement in strength and flexibility exercises was positively associated with life satisfaction (*B* = 3.203, SE = 0.854, *t* = 3.752, *p* < 0.001). Having two children (*B* = 3.498, SE = 1.100, *t* = 3.180, *p* = 0.002) or three or more children (*B* = 2.618, SE = 0.971, *t* = 2.697, *p* = 0.008) was also positively associated with life satisfaction relative to the reference group comprising participants with one or no children. Regarding educational level, primary education was not significantly associated with life satisfaction after adjustment for the other variables in the model (*B* = 1.271, SE = 0.752, *t* = 1.689, *p* = 0.093). In contrast, technical education showed a significant negative association (*B* = −4.744, SE = 1.597, *t* = −2.971, *p* = 0.003). Because primary and technical education were entered as dummy variables, their coefficients represent differences relative to participants in the remaining educational categories included in the reference group. Although mild food insecurity showed a positive coefficient (*B* = 1.526, SE = 0.776, *t* = 1.966), the association did not reach the conventional threshold for statistical significance (*p* = 0.051). Thus, although food insecurity was significantly associated with life satisfaction in the bivariate analyses, it did not independently predict life satisfaction after adjustment for the other variables included in the regression model.

## 4. Discussion

The present study examined factors associated with life satisfaction among older adults by integrating psychological, economic, family, food-related, and physical activity characteristics. The adjusted model showed that higher perceived stress, perceived insufficient income, self-employment, other sources of income, and technical education were associated with lower life satisfaction. Conversely, combined engagement in strength and flexibility exercises and having two or three or more children were associated with higher life satisfaction. These findings are broadly consistent with the conservation of resources theory, which proposes that well-being is influenced by the availability, preservation, and perceived threat of personal, social, and material resources [6,42].

Perceived stress showed a robust negative association with life satisfaction. Previous research in older populations has similarly documented an inverse relationship between perceived stress and life satisfaction and has highlighted the role of coping resources in this association [43]. From a conservation of resources perspective, persistent demands may undermine well-being when individuals perceive that the resources available to manage them are insufficient or threatened [42]. In later life, this process may become particularly relevant when psychological demands coexist with economic uncertainty, functional changes, or reduced access to social resources. Accordingly, the present findings suggest that the subjective appraisal of daily demands is an important component of how older adults evaluate their lives.

Economic conditions were also independently associated with life satisfaction. Participants who perceived their income as insufficient reported lower life satisfaction, supporting evidence that subjective financial security is closely related to subjective well-being [14,15,44]. Financial well-being involves not only the amount of available income but also perceptions of stability, security, and capacity to meet current and future needs. These perceptions may be particularly consequential in later life, when opportunities to replace lost income can be limited and health- or care-related expenses may become more salient.

Self-employment and other sources of income were also negatively associated with life satisfaction relative to pension income. These findings should be interpreted cautiously because the study did not assess income amount, regularity, employment conditions, or social protection. Nevertheless, the pattern may indicate that the predictability and perceived security of economic resources are relevant to well-being, rather than source of income alone. This interpretation is compatible with evidence showing that subjective financial security is associated with life satisfaction among older adults [44]. Future studies should therefore distinguish income level from income stability, employment necessity, pension adequacy, and perceived financial strain.

In contrast, engaging in both strength and flexibility exercises was positively associated with life satisfaction. This finding is consistent with evidence linking physical activity and functional fitness with better life satisfaction, autonomy, and quality of life in older adults [22,45]. Strength and flexibility are particularly relevant to the preservation of mobility and the capacity to perform everyday activities. Within the conservation of resources framework, maintaining physical function may constitute an important personal resource because it supports independence and perceived control. However, given the cross-sectional design, the association may also be bidirectional: older adults with greater well-being or functional capacity may be more likely to engage in these activities.

Having two or three or more children was positively associated with life satisfaction compared with the combined reference category of having one or no children. Although previous research has linked family and social support with greater life satisfaction in older populations [46,47], the number of children should not be interpreted as a direct measure of available support. Family relationships can provide companionship, practical assistance, emotional closeness, and a sense of belonging, but their contribution to well-being depends on relationship quality and the type of support exchanged. Therefore, the present result may reflect differences in potential family resources, but this mechanism cannot be established because perceived support, frequency of contact, and relationship quality were not directly assessed.

The findings regarding educational level require particular caution. Primary education was not significantly associated with life satisfaction after adjustment, and the bivariate post hoc analysis likewise showed no significant difference between participants with primary and high school education. Consequently, the corrected results do not support the previous interpretation that primary education may confer an advantage in life satisfaction through lower expectations or adaptation. This clarification is important because educational attainment should not be interpreted as having a simple linear relationship with subjective well-being.

Technical education, in contrast, showed a negative association with life satisfaction. However, this result should be regarded as exploratory. The technical education subgroup was small (n = 10), and its dummy coefficient represents a comparison with a combined reference group comprising other educational categories. The estimate may therefore be sensitive to the distribution of participants across categories or to unmeasured characteristics related to occupational history, lifetime socioeconomic conditions, or retirement circumstances. Replication in larger samples with more balanced educational groups is needed before attributing substantive meaning to this association.

Food insecurity displayed a clearer pattern in the bivariate analyses than in the adjusted model. Participants with mild food insecurity reported higher life satisfaction than those with moderate or severe insecurity; however, the corresponding dummy variable did not reach statistical significance after adjustment. This attenuation may indicate shared variance with perceived income sufficiency and stress. Food insecurity has been associated with poorer mental health among older adults in low- and middle-income countries and with poorer quality of life in older populations [16,18]. Rather than suggesting that food insecurity is irrelevant, the present findings indicate that its association with life satisfaction may be embedded within a broader context of economic and psychological vulnerability. Longitudinal or mediation models would be needed to evaluate these pathways directly.

Depression and anxiety were also negatively correlated with life satisfaction, while both were positively associated with perceived stress. This pattern is consistent with evidence linking emotional distress with less favorable evaluations of life [23]. Nevertheless, the final regression model included perceived stress as the psychological predictor; therefore, the present analyses do not establish whether stress has a stronger independent contribution than depression or anxiety. Future studies that simultaneously model these constructs could determine whether their associations with life satisfaction are independent, overlapping, or mediated through a broader psychological distress pathway.

Overall, the results support a resource-based and multidimensional interpretation of life satisfaction in later life. Psychological appraisal, perceived economic security, functional physical activity, and potential family resources were independently associated with life satisfaction, whereas the bivariate association with food insecurity was attenuated after adjustment. This pattern is consistent with the broader concept of healthy aging, which emphasizes functional ability and the capacity to live in accordance with what individuals value rather than the absence of disease alone [5]. The findings therefore reinforce the importance of examining subjective well-being within the social, economic, psychological, and functional context in which aging occurs.

### 4.1. Implications

The findings suggest several areas for community and primary care practice. Perceived stress may be useful as part of brief psychosocial assessments of older adults, particularly when psychological distress coexists with economic or functional difficulties. Screening should be accompanied by access to appropriate support, including strategies to strengthen coping resources, social connections, and referral pathways when clinically relevant.

The association between combined strength and flexibility exercise and life satisfaction also supports the incorporation of functional physical activity into healthy aging programs. Interventions delivered through community centers, health services, or municipal programs could prioritize safe and adapted activities aimed at preserving strength, mobility, balance, and flexibility. Such approaches are consistent with healthy aging strategies focused on maintaining functional ability and autonomy.

Economic vulnerability should also be considered within well-being initiatives for older adults. The associations of perceived insufficient income and non-pension income sources with lower life satisfaction suggest that assessments based solely on income amount may overlook subjective financial insecurity. Community and social services may benefit from identifying difficulties related to meeting basic needs, access to pensions or social benefits, and instability of available resources.

Finally, family size should not itself be treated as an intervention target. Rather, the association observed with number of children points to the potential relevance of relational resources. Assessment of perceived social support, loneliness, family relationship quality, and community connections would provide more actionable information than the number of relatives alone. Similarly, the bivariate association observed for food insecurity supports continued screening for food-related vulnerability, particularly when it occurs alongside financial strain or psychological distress.

### 4.2. Limitations

Several limitations should be considered. First, the cross-sectional design precludes conclusions about temporality or causality. Terms such as “predictor” refer to statistical prediction within the regression model and should not be interpreted as evidence that the identified variables cause changes in life satisfaction. Reverse or reciprocal relationships are also plausible.

Second, participants were recruited through nonprobability convenience sampling in a single municipality. Consequently, the findings may not generalize to all older adults in Sogamoso or to other Colombian or Latin American populations. The use of an online questionnaire may have further favored individuals with greater digital literacy, Internet access, functional independence, or social connectivity.

Third, the PHQ-2, GAD-2, and PSS-4 are brief self-report measures intended primarily for screening or population research rather than clinical diagnosis. The comparatively modest internal consistency observed for the PHQ-2 and PSS-4 in the present sample should also be considered, as measurement error may attenuate associations. In addition, all measures were self-reported and may therefore be affected by recall, interpretation, or social desirability.

Fourth, several categories contained relatively few participants. This is particularly relevant to technical education and strength-only exercise, for which coefficient estimates may be less stable. Moreover, the dummy coding strategy used in the regression means that some coefficients compare a specific category with a combined reference group. They should therefore not be interpreted as establishing an ordered gradient across all categories.

Fifth, potentially relevant determinants of life satisfaction were not included, such as chronic disease burden, pain, frailty, objective physical functioning, sleep quality, loneliness, perceived social support, relationship quality, community participation, income amount, and financial stability. Their inclusion could clarify some of the associations observed, particularly those involving number of children, source of income, and physical activity.

Finally, the correlations among depression, anxiety, and perceived stress suggest that psychological distress encompasses related but distinguishable experiences. Because the final regression did not simultaneously estimate the independent contributions of all three variables, future studies could use longitudinal or structural equation models to examine their unique and shared pathways together with economic conditions, food insecurity, social resources, and physical functioning.

## 5. Conclusions

Life satisfaction among the older adults studied was associated with psychological, economic, family, and functional factors. Higher perceived stress, perceived insufficient income, self-employment, and other sources of income were associated with lower life satisfaction, whereas combined strength and flexibility exercise and having two or three or more children were associated with higher scores. Food insecurity showed a significant bivariate relationship with life satisfaction but did not remain statistically significant after adjustment.

The corrected analysis did not support an association between primary education and greater life satisfaction. The negative coefficient observed for technical education should be regarded as exploratory because of the small subgroup and the dummy-coding structure of the model. Overall, the results support an integrated approach to subjective well-being in later life that considers psychological appraisal, economic security, functional capacity, and relational resources.

Future research should prioritize longitudinal and probability-based designs, combine digital and face-to-face recruitment, and incorporate more detailed measures of social support, physical functioning, financial security, and health status. Such approaches could clarify the mechanisms through which these resources jointly shape life satisfaction during aging.

## Figures and Tables

**Table 1:** Comparison of life satisfaction scores according to sociodemographic characteristics, physical activity, and food insecurity.

Characteristics	Category	n	%	M ± SD	t/*F*	*p*
Sex	Female	71	35.5	23.44 ± 5.81	t = −1.26	0.209
	Male	129	64.5	24.57 ± 6.59		
Area of residence	Rural	33	16.5	25.55 ± 6.27	t = 1.38	0.175
	Urban	167	83.5	23.90 ± 6.33		
Sufficient income	No	98	49.0	21.34 ± 5.96	t = −6.87	<0.001 ***
	Yes	102	51.0	26.89 ± 5.46		
Age	60–69 years	126	63.0	24.02 ± 6.43	*F* = 0.28	0.753
	70–79 years	56	28.0	24.18 ± 6.42		
	80 years or older	18	9.0	25.22 ± 5.58		
Educational level	High school	57	28.5	23.11 ± 6.82	*F* = 5.91	<0.001 ***
	Primary education	89	44.5	23.28 ± 5.91		
	Technical education	10	5.0	24.00 ± 8.08		
	University	27	13.5	25.41 ± 5.09		
	Graduate studies	17	8.5	30.53 ± 3.43		
Marital status	Married	109	54.5	25.78 ± 6.24	*F* = 5.24	<0.001 ***
	Divorced	27	13.5	21.44 ± 7.12		
	Single	33	16.5	21.24 ± 5.73		
	Cohabiting	14	7.0	23.43 ± 5.15		
	Widowed	17	8.5	24.47 ± 4.30		
Number of children	1	16	8.0	21.44 ± 6.50	*F* = 2.13	0.098
	2	48	24.0	25.31 ± 5.82		
	3 or more	121	60.5	24.34 ± 6.49		
	None	15	7.5	22.07 ± 5.68		
Type of income	Employee	18	9.0	22.17 ± 6.09	*F* = 9.54	<0.001 ***
	Self-employed	84	42.0	23.98 ± 6.08		
	Other	40	20.0	21.03 ± 6.16		
	Pensioner	58	29.0	27.24 ± 5.61		
Aerobic activity	Active	100	50.0	25.44 ± 6.40	*F* = 2.37	0.054
	Underactive	6	3.0	24.83 ± 7.57		
	Regular underactive	51	25.5	23.16 ± 6.26		
	Light regular underactive	21	10.5	22.76 ± 5.15		
	Sedentary	22	11.0	21.91 ± 6.14		
Strength and flexibility	Both	43	21.5	27.65 ± 5.37	*F* = 8.00	<0.001 ***
	Strength only	6	3.0	18.83 ± 3.06		
	Flexibility only	49	24.5	24.49 ± 5.65		
	Neither	102	51.0	22.86 ± 6.54		
Food insecurity	Mild	131	65.5	25.65 ± 5.59	*F* = 11.48	<0.001 ***
	Moderate	51	25.5	21.49 ± 6.41		
	Severe	18	9.0	21.00 ± 7.82		

Note: Welch’s independent-samples *t*-test was used for comparisons involving two groups, and one-way ANOVA was used for comparisons among three or more groups. Tukey’s HSD test was used for post hoc comparisons following statistically significant ANOVAs. M = mean; SD = standard deviation. *** *p* < 0.001.

**Table 2:** Means, standard deviations, and correlations between psychological variables and life satisfaction.

Variable	M	SD	1	2	3	4
1. Depression	2.32	1.81	—			
2. Anxiety	1.98	1.92	0.57 **	—		
3. Perceived stress	5.79	3.20	0.52 **	0.71 **	—	
4. Life satisfaction	24.17	6.34	−0.31 **	−0.41 **	−0.52 **	—

Note: M = mean; SD = standard deviation. ** *p* < 0.01.

**Table 3:** Multiple linear regression predicting life satisfaction.

Variables	B	SE	t	*p*
Intercept	27.398	1.492	18.362	<0.001 ***
Perceived stress	−0.762	0.112	−6.805	<0.001 ***
Number of children: 2 ^a^	3.498	1.100	3.180	0.002 **
Number of children: 3 or more ^a^	2.618	0.971	2.697	0.008 **
Educational level: primary education ^b^	1.271	0.752	1.689	0.093
Educational level: technical education ^b^	−4.744	1.597	−2.971	0.003 **
Income: employee ^c^	−2.054	1.318	−1.559	0.121
Income: self-employed ^c^	−2.153	0.814	−2.646	0.009 **
Income: other ^c^	−2.569	1.063	−2.417	0.017 *
Sufficient income: no ^d^	−3.380	0.774	−4.366	<0.001 ***
Food insecurity: mild ^e^	1.526	0.776	1.966	0.051
Physical activity: strength and flexibility ^f^	3.203	0.854	3.752	<0.001 ***

Note: Dependent variable = life satisfaction. N = 200. B = unstandardized regression coefficient; SE = standard error. Dummy variables were coded as follows: ^a^ The reference group comprised participants with one or no children; ^b^ The reference group comprised the remaining educational categories (high school, university, and graduate studies); ^c^ Pensioner was the reference category; ^d^ Sufficient income (“yes”) was the reference category; ^e^ Moderate or severe food insecurity constituted the reference group; ^f^ Participants not simultaneously engaging in both strength and flexibility exercises constituted the reference group. *F*(11, 188) = 16.23, *p* < 0.001; *R*^2^ = 0.487; adjusted *R*^2^ = 0.457. Statistical significance levels: * *p* < 0.05; ** *p* < 0.01; *** *p* < 0.001.

## Data Availability

The data presented in this study are available on request from the corresponding author. The data are not publicly available because they contain information derived from human participants, and public deposition could compromise participant confidentiality and privacy.
